# British Lying-In Hospital

**Published:** 1830-03

**Authors:** 


### BRITISH LYING-IN HOSPITAL.

### *Uterine Phlebitis, with Ulceration of the Articular Cartilages, and Purulent Effusion within the Capsular Ligament of the right Knee-joint.**

Mrs. Mayhew, ætatis thirty-three, was delivered in the British Lying-in Hospital, on the 2d March, 1829, after an easy and natural labour. The placenta was expelled in a few minutes after the infant, and she appeared to recover in a favorable manner, until the third day, when a discharge of blood from the uterus took place.

From the 6th to the 20th of March, she made no complaint of uneasiness in any region of the body, though the strength rapidly declined. The countenance was of a dusky yellow hue; the heat of the surface slightly increased; the respiration hurried, particularly on bodily exertion, and the pulse was above 130, and feeble; the tongue pale and glossy, with total loss of appetite, though at no period was there nausea or vomiting. The uterus gradually receded into the pelvis, and pressure over the hypogastrium produced no sensible uneasiness. The milk was secreted sparingly. The lochial discharge had a peculiarly offensive smell.

From the 20th to the 28th, when she died, the prostration of strength increased, and the pulse became still more frequent and feeble; the respiration was extremely hurried, and she was incessantly harassed with a hacking cough, and the expectoration of a frothy mucus. The abdomen continued soft and flaccid, and not affected by pressure. She, however, during this period, complained of excruciating pains in all the joints of the right superior extremity, and in the right knee-joint, which was observed to be considerably swollen, but not discoloured.

*Dissection.* The uterus was found reduced to its usual size a month after delivery, and no morbid appearance was visible on its peritoneal surface, except a slight adhesion between its left margin, near the entrance of the fallopian tube and the rectum. The uterus being removed from the body, and the cavity laid open, there was found to be a portion of placenta, about the size of a nutmeg, in a putrid state, adhering to its inner surface, at the part corresponding with the adhesion between the peritoneal coat and rectum. The substance of the uterus, to the extent of an inch around this, was of a peculiarly dark colour, almost black, and as soft as sponge. On cutting into it, about a teaspoonful of puru-

* From a paper of Dr. R. Lee's, "on Inflammation of the Veins of the Uterus, &c." Medico-Chir. Trans. vol. xv. part ii.

lent matter escaped from the veins, and a small additional quantity was pressed out from them. The spermatic and other abdominal veins presented no morbid appearance, and the uterine appendages were healthy.

On opening the capsular ligament of the right knee-joint, where a fluctuation was perceived, about six ounces of thin purulent fluid escaped, and the cartilages of the femur and tibia were extensively eroded. There was no appearance of inflammation, however, exterior to the capsular ligament. The right wrist was swollen, but the structure of the joint was not affected. The cellular membrane around it was unusually vascular, and infiltrated with serum.

The patient, whose case I have now related, was under the immediate care of my friend and colleague Dr. Henry Davies, who was present at the dissection, and also Mr. Armstrong, of Golden square.*

The preceding case, and others of a similar description, to which Mr. Arnott and M. Dance have alluded in their valuable essays, induced me to believe that the violent and destructive affections of the joints, which sometimes occur in puerperal women, were invariably connected with inflammation of the uterine veins; but in the following fatal cases, though these vessels were minutely examined, no morbid alteration of structure in their coats could be detected.†

---

### *Inflammation of the Saphena Veins, fatal on the sixteenth day after Parturition.*‡

Mrs. Mills, æt. thirty, a patient of the British Lying-in Hospital, was delivered of her fourth child on the 7th instant, after a natural labour. During the latter months of gestation, she had suffered much from œdema, and a varicose state of the veins of the lower extremities. Two days after her confinement, she began to complain of pain in the superficial veins of both legs; and, during the subsequent weeks, a diffuse swelling and erysipelatous redness of the surface took place in the calf of the left leg, and in a less degree in that of the right. This was accompanied with violent febrile disturbance.

I first saw her on the 16th instant, the seventh day after the commencement of the disease. The pulse was 100; tongue red;

* See Medical Gazette, vol. iii. April 25, 1829.

† Dr. Denman had noticed these affections of the joints in puerperal women, for he observes, "There is a peculiarity in this fever, which I believe has not hitherto been observed or mentioned. It is an erysipelatous tumor, of a dusky red colour, on the knuckles, wrists, elbows, knees, or ankles, about the size of a shilling, and sometimes larger. This is almost universally a mortal sign, and, on the inspection of those who have died with this appearance, the disease has been found to have affected principally the uterus or its appendages." (Introduction to Midwifery, p. 570.)

‡ Ibid.

countenance flushed, skin hot, and respiration hurried, with much jactitation and delirium.

The left lower extremity, now chiefly affected, presented the following appearances: From the knee to the ankle, on its inner surface, the integuments were hot, swollen, and tense; and in several places, large patches of a dark red colour were observed over the veins; which being laid open in two places, a considerable quantity of purulent fluid was discharged. Where the swelling and tension were least, the superficial veins could be felt distended like hard cords, as could also the saphena, through its whole course upward from the ham to its junction with the femoral vein. In the course of this vein there was considerable swelling, and the integuments in this situation, as far as the middle of the thigh, were hot, and of a dark red colour.

The right leg was similarly affected, but in a very inferior degree to the left.

17th October.—Pulse 120. Little marked change in the general symptoms. Left thigh much more swollen, and the saphena vein now painful, indurated, and enlarged. Above the ankle other two abscesses have formed, and been opened. A small abscess has also formed above the knee of the right extremity, which in other respects is improving.

19th.—The left extremity, from the ankle to the groin, is on its surface more swollen and painful, and the saphena vein can be felt still more enlarged. The abdomen is tympanitic and exquisitely sensible on the left side when pressed. Pulse 160; subsultus tendinum; urgent thirst; tongue brown and parched; skin hot; countenance flushed and anxious; delirium diminished.

During the succeeding three days, there was a gradual exacerbation of all the symptoms, and she died on the 23d instant, being the fourteenth day from the commencement of the symptoms.

My friend Dr. Sims was present, and assisted me at the dissection on the 24th, and the following is our joint report of the morbid appearances:

The extremity was very much enlarged. The cellular and adipose membranes from Poupart's ligament, along the inner surface of the thigh and leg to the ankle, were indurated, vascular, and infiltrated with a red-coloured serous fluid. Several abscesses were observed in the cellular tissue immediately beneath the skin in the calf of the leg, and an extensive collection of purulent fluid had formed in the interstices of the gastrocnemii muscles. The branches of the saphena vein in this situation were converted into solid impervious cords, and the coats of this vein from the knee to its junction with the femoral were thickened and contracted; and in the lower part the cavity was nearly obliterated. The saphena vein was lined with an adventitious membrane of considerable thickness, which was easily separated from the inner coat. Its opening into the femoral vein, though reduced in size, was pervious, and the coats of the femoral from this point to the ham were thickened and contracted. The inner membrane was rugous, and of a deep red colour, but no deposit of lymph was observable, and its canal was pervious.

The femoral vein above the termination of the saphena, and the whole of the external iliac, were thickened, and slightly contracted in their diameters, and lined with a thin coating of lymph. These vessels were pervious, and the common and internal iliac exhibited no sign of disease.

The intestines were inflamed, and on the ascending colon there was a small part in a state of sphacelus.

---

